# Fast and slow health crises of Homo urbanicus: loss of resilience in communicable diseases, like COVID-19, and non-communicable diseases

**DOI:** 10.1097/j.pbj.0000000000000073

**Published:** 2020-07-17

**Authors:** Tari Haahtela, Josep M. Anto, Jean Bousquet

**Affiliations:** a Skin and Allergy Hospital, Helsinki University Hospital, University of Helsinki, Helsinki, Finland; b ISGlobal, Centre for Research in Environmental Epidemiology (CREAL), Barcelona, Spain; c Charité. Charité, Universitätsmedizin Berlin, Humboldt-Universität zu Berlin, and Berlin Institute of Health, Comprehensive Allergy Center, Department of Dermatology and Allergy, Berlin, Germany; d University hospital, Montpellier, France.

New kind of health hazards awaits the present and future human populations. Considering the COVID-19 pandemic, much of the problems may arise from the loss of resilience, which has been defined as an ability to recover from or adjust to misfortune or change (Merriam-Webster Dictionary). The concept of resilience is complex and multidimensional, and psychological, societal, and immunological aspects are all critical to cope with a pandemic. Lack of the resilient immunity at individual and community level is obvious as COVID-19 seems to be a new virus to humans.

What is also new is the massive and continuous information flow to which we are exposed as never before. We experience a global panic in the era of digital and global information, spreading out in a second. A new concept, *infodemic*, as a continuum to epidemic and pandemic, may describe this phenomenon. We are used to deal with various *risk factors* but we also need to explore and test *protective factors.* What are the determinants that make individuals and communities resilient against fast and slow health crises like outbreaks of infectious diseases and chronic noncommunicable diseases (NCDs) (Fig. 1)?

**Figure 1:**
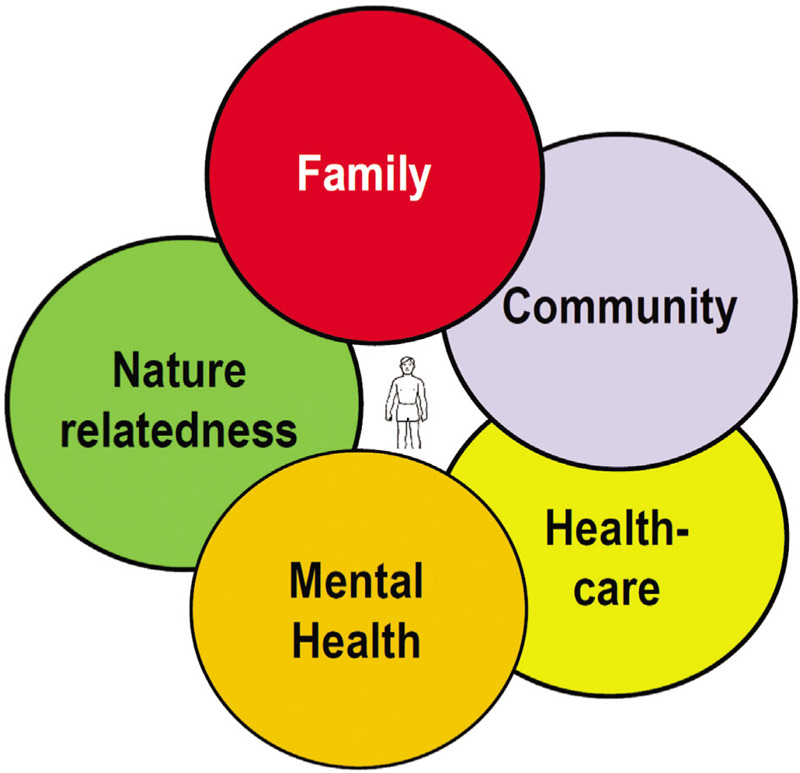
Both individual and community determinants create resilience in health crises.

As the community resilience refers to the sustained ability to respond and to recover from shocks and stressors, it is becoming of outmost importance to make a difference between the true truth (ie, facts based on valid science) and the fake truth. It is good news that this may mean an uprise of evidence-based science and factful information in the modern time of “post-truth.”

## Personal notes (TH)

I am 74 years of age, and began my career by taking care of patients with tuberculosis in Finland. In 1930, almost 10,000 citizens in Finland died every year because of tuberculosis. At that time, the Finnish population was around 4 million. Almost half of the diseased patients died, and a significant part of them were young adults. The poor Nordic country built 17 massive sanatoriums to fight the scourge. Gradually, a comprehensive system was built: mass-miniature x-rays to find the early cases, Calmette vaccinations, sanatorium care, rehabilitation…, and finally the life-saving new drugs. Until May 2020, some 260 patients have died in Finland, because of the COVID-19. During a “normal” year, around 500 patients die because of seasonal influenza, without a big notice.

I have lived through the Asian influenza 1957 to 1958 (at least 1 billion had it and maybe 2 million died), the Hong Kong pandemic 1968 to 1969 (900 million were diseased and almost a million died). At that time, I was a medical trainee in the Helsinki University Hospital, and still remember some of the patients, for example, those with asthma exacerbations. Then, we had SARS-COVID in early 2000s, but it did not really reach Finland.

When I was growing up in Helsinki in 1950s, there were no vaccinations or medications against measles, rubella, chickenpox, parotitis, smallpox, or polio. When our neighbor got chickenpox, my mother took me and my brother there to get the infection before puberty as it can cause infertility as a complication.

All this seems more or less forgotten or ancient history. Yet, it happened almost yesterday!

## Historical notes

Population growth and decrease in mortality in Europe started with the new agricultural practices in the 18th century. Industrial revolution in 19th century was associated with sharp decline of fertility and decrease in family size. In 1950s the capitalism led to the *great acceleration* which coincides with *Anthropocene*, a title suggested for a geological epoch of human impact on Earth's ecosystems.^1^

Large malnourished populations in medieval age and later on were extremely vulnerable to epidemics. Even 1918 influenza could be attributed to this pattern. This preceded the successful fight against infection epidemics, which only fully started in 1950s. Antibiotics, vaccinations, improved hygiene, and other medical, technical, and scientific achievements have contributed to the population explosion, the increase in life expectancy, and the aging.

After 1950s, the great acceleration, which had positive impacts for health in high-income countries, had profound negative consequences on the *health of planet*. Escalating urbanization, overuse of natural resources, and uncontrolled migration of populations became the rule. The increase in emissions of CO_2_ and other greenhouse gases, the global warming, and the massive extinction of species are all part of the *Anthropocene*. The evolutionary law of *natural selection* is not working as before. As a result, we are losing resilience as individuals and populations. We face epidemics of both *communicable* diseases (fast) and NCDs (slow). The outcomes are quite unpredictable.

## Dawn of noncommunicable diseases

When the tuberculosis epidemic slowed down in Finland, the sanatoriums had room to take care of also other conditions. Asthma and allergies drew attention as their occurrence seemed to be on rise.

Indeed, they are good indicators of the modern health hazards, for example, as shown in the Finnish and Russian Karelia.^2^ The disease pattern of those populations were quite contrast. In a subpopulation, in which the gene activation and its microbial determinants were explored, sensitization to common pollen allergens was 35% in the Finnish and 7% in the Russian young people (IgE >0.35 kU/L). Food allergies were almost nonexistent in Russian Karelia, and peanut allergy unknown. The contrast is neither explained by hereditary factors nor by air pollution or common chemicals. It is explained by changes in natural environment and lifestyle. In a relatively short period of time, after the Second World War, 2 geoclimatically and genetically close populations have developed quite contrasting immunological expression.

Data from 1920s to 2000s of asthma and allergies in Finnish conscripts (men, 18–19 years of age) indicated a rise starting in those born after the Second World War.^3^ The allergy “epidemic” became slowly apparent in 1960s, when the young men born in 1940s approached the military conscription age. This is true also for obesity, diabetes, hypertension, depression, and inflammatory bowel diseases.

In the early 2000s, it was acknowledged in Finland, that the strict avoidance strategy of allergens and irritants has not stopped the allergy rise. The paradigm was turned around, and a real life act to improve tolerance/resilience was taken in the Finnish Allergy Programme (2008–2018).^4^ Connections to natural environments and its microbiota was emphasized (eating, drinking, breathing, and touching). Health care professionals were educated and lay-public informed. The results are promising as morbidity and costs started to reduce.^5^

The epidemic of allergy is not an isolated case. It is concurrent with the epidemic increase of obesity, cardiovascular diseases, mental diseases, and other chronic inflammatory disorders. Even cancer has been connected to this trend.^6^ The experience of the Finnish Allergy Program may show the way for other similar attempts to mitigate the burden of NCDs.^7^

## The technosystem as a friend and foe

Global Burden of Disease is used as a measure of population health, but even more importantly it may indicate disconnection from natural environments, soil, and natural waters, the evolutionary home of man. Mid-Victorian people in the United Kingdom (1850–1880) were less urbanized, and life expectancy at age 5 was as good or better than that exists today.^8^ The disease pattern was quite different lacking much the NCDs.

The human technosystem invents tools to improve resilience, but only real life can test their usefulness over long periods of time. Digital era has hugely speeded up obtaining and spreading information, and connected human cognitive capacity together as never before.^9^ Identifying and realizing global threats have improved tremendously. For a significant part of human kind, better life and health standard have been achieved. In fact, we may be living the best historical period ever.^10^

But the technosystem has also created risks as never seen before. The immediate ones are directly associated with our digital era. Chinese government implemented strict measures to control COVID-19 epidemic, and tightened the control to extremes. For the first time in human history, a kind of *digital totalitarianism* was used to stop the epidemic. The risks of this approach are obvious, if digital surveillance is employed for political aims.^11^ What happens when the new technology serves certain political agendas, to discriminate, suppress, and even destroy? The latter has taken place in recent past, even without any digital armamentarium.

Science and culture should guide the way technology is used and implemented for the best of development and equitable societies. Not surprisingly, populist governments are deploying technological strategies while coercing the independence of science and the cultural and political dissent.

## The many faces of resilience

Resilience is central not only to individuals but to societies and populations by and large.^12^ It is strengthened by an environment of multiple options, which gives room for choices. If one option fails, the other may succeed. In a biological or psychological monoculture, the failure of one may mean a failure of all. Biodiversity denotes abundance and even waste instead of extreme effectiveness. Paradoxically, waste creates buffers, i.e. resilience. If a population is homogenous in the lack of immunity against COVID-19, it may face extinction.

The Planetary Health concept was inspired by the paradox that during the great acceleration period, traditional metrics for human health were improving as the main planetary metrics were showing major negative impacts.^13^ During this period of some 70 years, an unexpected outcome may have been loss of human health resilience, which becomes apparent during the pandemics. If biological resilience is weakening, the ability to adapt is at stake.

The fuel for immunological resilience is exposure to biodiverse life.^14^,^15^ Human interaction with biological macro- and microdiversity is decisive, but social and political diversity are equally important. Because natural biodiversity in human environments has been seriously reduced, so has our immune resilience. Urban populations are short of experience, especially introduced by microorganisms. The same may have happened with our mental resilience. The psychological challenges and experiences we have met during the million years of evolution, and coped with, are essential for us to survive.^16^,^17^ The profound individual and societal changes of the great acceleration period may have profoundly affected our capacity to deal with stress and shocks.

**Figure 2:**
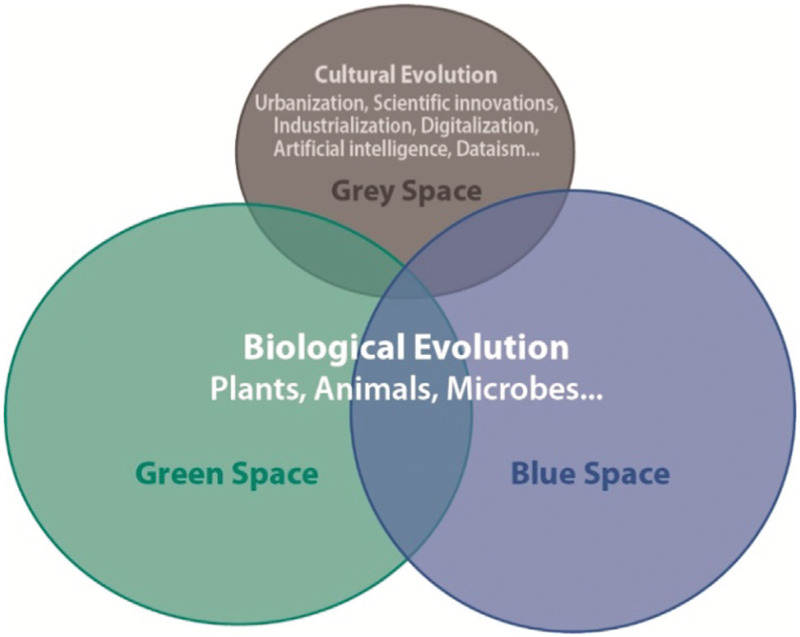
Human kind has evolved from natural environments, that is, from green (soil) and blue (waters), but is increasingly affected by cultural environment, that is, grey (urban) space.^15^

The scientific progress has created *cultural and technological evolution* (technosystem) built on the *biological evolution* (ecosystem). Science and cultural evolution may create life 2.0, but it lacks the prerequisites of sustainability (Fig. 2).

The UN Agenda 2030 has been aimed at strengthening individual-, community-, and system-level resilience for population health and well-being.^18^ Understanding, restoring, and strengthening resilience is crucial in order to make progress toward the implementation of the Sustainable Development Goals.

## Fast and slow health crises

Resilience is key to face both fast and slow health crises. Pandemics are a paradigm of fast health crises. COVID-19 is a serious and even fatal scourge, and should not be underestimated. We must reduce and stop the trade of wild caught animals and ban the sale of these animals in wet market, as the virus may have originated from bats.^19^ Altogether, respecting and safeguarding biodiverse, natural environments are crucial for human kind to survive. Nevertheless, the zoonoses have always been there, and the risks must be put into a perspective to avoid even bigger catastrophes.^20^ COVID-19 pandemic causes huge economical losses, which are magnified by the global panic. Closing up societies leads to economical depression, which is painfully reflected in population health, especially of the old, poor, and less privileged.

With COVID-19, the lack of immunological resilience is obvious, and reach for an effective vaccination programme central. But loosing psychological resilience has led to a profound change in mind set. In long-term, that may have even more serious adverse effects than the pandemic itself.

Slow health crises are a persistent trend. The epidemic growth of NCDs is a slow crisis. It gives more time for adaptive changes, but there are thresholds and turning points that could eventually result in acute disruptions. Global warming due to anthoropogenic earth changes is our main slow crisis. For the Intergovernmental Panel on Climate Change panel, however, deep understanding of the climate systems and the risk of tipping points have led to formulate even extreme urgency for the goals.^21^

Our civilization is a complex system deeply rooted in the Earth conditions that make it possible to develop and evolve. With COVID-19 we are facing a major fast crisis. This crisis came in a context of unprecedented slow crises like NCDs, global warming, and biodiversity loss. It may even turn out that the virus spread among humans because the biodiverse environments of bats, the natural hosts of the virus, were destroyed.

We face the *butterfly effect*: a butterfly flapping its wings on one side of the world causes chaos on the other side. Butterflies are unlikely to be affected by coronaviruses, but their flapping effects may turn to be the last opportunity for us to reconcile our civilization with the nature. As we say in Finland, we should go along nature, not against it.

## Conflicts of interest

The authors report no conflicts of interest.
